# Prevalence of Human Papillomavirus (HPV) Genotypes among Women During 2015–2020 in Mashhad, Iran

**DOI:** 10.34172/aim.2023.64

**Published:** 2023-08-01

**Authors:** Anis Bakhshani, Rashin Ganjali, Seyed-Elias Tabatabaeizadeh

**Affiliations:** ^1^Cellular and Molecular Biology Department, Razi University of Kermanshah, Kermanshah, Iran; ^2^Norouzpour Pathobiology Laboratory, Mashhad, Iran; ^3^Immunology Research Center, Bu-Ali Research Institute, Mashhad University of Medical Sciences, Mashhad, Iran; ^4^Mashhad Branch, Razi Vaccine and Serum Research Institute, Agricultural Research, Education and Extension Organization (AREEO), Mashhad, Iran

**Keywords:** Cervical cancer, Genotyping, Human papillomavirus, Iran, Liquid base cytology

## Abstract

**Background:**

Cervical cancer is the fourth most common cancer in women, and human papillomavirus (HPV) is the leading cause of cervical cancer. Cervical cancer screening and HPV vaccination are important in the incidence of cervical cancer.

**Methods:**

This study was performed on Liquid Base Cytology (LBC) samples of 1214 women in Mashhad who were referred for cervical cancer screening in 2015-2020. Samples were examined by Single-Step PCR and Reverse Line Blot for HPV genotyping.

**Results:**

386 women (31.8%) were HPV PCR positive. HPV genotyping of 277 samples showed that HPV 31 (3%), 16 (2.5%), 51 (2.2%), 18 (2%), and 66 (1.8%) were the most prevalent high-risk HPV (hrHPV) genotypes. Among low-risk HPV (lrHPV) genotypes, HPV 6 (9.2%), 53 (4.7%), and 42 (2.8%) were the most common genotypes. The range of multiple infections varied between two to eight genotypes and the prevalence of multiple HPV infections (12.4%) was higher than single infections (10.4%). For women with single HPV infections, HPV 31 and 66 were equally the most common hrHPV genotypes, followed by HPV 16 and 39. In women with multiple HPV infections, HPV 31 was the most common hrHPV genotype, followed by HPV 51 and 16. For both the single and multiple HPV infections, HPV 6 was the most common lrHPV genotype, followed by HPV 53 and 42.

**Conclusion:**

In conclusion, due to the high prevalence of HPV single and multiple infections, the need for governmentally supported HPV vaccination and through cervical cancer screening should be emphasized to prevent cervical cancer.

## Introduction

 Globally, cervical cancer is the fourth most prevalent cancer in women after breast, colorectal, and lung cancers, with 570 000 cases of cervical cancer and 311 000 deaths from the disease in 2018.^1^ The estimated annual age-standardized incidence rate (ASIR) is 15 per 100 000 women globally; however, Iran is among the 12 countries located in western Asia or the western part of central-south Asia with the lowest ASIR values ( < 5 per 100 000 women).^1^

 Human papillomavirus (HPV) vaccination and cervical cancer screening play an important role in the incidence of cervical cancer. Accordingly, cervical cancer is the second most frequent cancer among women in developing countries, where the levels of HPV vaccination and screening for cervical cancer are significantly lower than those in developed countries.^2,3^ HPV causes almost all cases of cervical cancer and some cancers of the vulva, vagina, oropharynx, and anus.^4^

 There are more than 200 known types of HPV, of which about 40 types can infect the genitals, making it the most common sexually transmitted infection in the world.^5^ HPV genotypes are categorized as high-risk HPV (hrHPV) and low-risk HPV (lrHPV) based on the possibility of being carcinogenic. The hrHPV genotypes include HPV 16, 18, 31, 33, 35, 39, 45, 51, 52, 56, 58, 59, 66, and 68; among them, HPV 16 and 18 genotypes cause 70% of cervical cancers and a high proportion of oropharyngeal and anogenital cancers.^6^ The lrHPV genotypes include HPV 6, 11, 42, 43, 53, 73, 81, 82, and 83; among them, HPV 6 and 11 cause more than 90% of benign genital warts.^6^

 Studies have shown that hrHPV testing has significantly improved cervical cancer detection compared to cytology.^7-9^ Accordingly, for primary screening, hrHPV testing is the method of choice in a number of developed countries.^10^

 Although several studies have been performed in different provinces and cities of Iran, the available data are still not enough, and comprehensive studies, like the present study, are required to provide a picture of infection with HPV genotypes in Iran.^11-15^ To this end, in this study, by examining an extensive panel of HPV genotypes over a period of five years, we tried to provide guidance for cervical cancer prevention and HPV vaccination in the country.

## Materials and Methods

###  Study Population and Sampling

 This study was performed on 1214 women who were referred to the Dr. Norouzpour Pathobiology Laboratory (DNPL) for HPV infection screening of liquid base cytology (LBC) samples from April 2015 to March 2020. The women were mostly urban and belonged to the middle class economically. Patient information analysis was undertaken in full compliance with the Helsinki Declaration, including anonymity and confidentiality. The age range was 24–60 years.

 DNPL is one of the independent laboratories located in Mashhad city. Mashhad is the capital of the Khorasan Razavi province and is located in northeastern Iran. After Tehran, the capital of Iran, Mashhad is the most populous city in the country. According to the latest information published by the Statistics Center of Iran in 2016, the total population of Mashhad was 3 001 184, of whom 1 497 360 were women. The tomb of the eighth Imam of the Shi’ite Muslims is in Mashhad, and millions of pilgrims travel to Mashhad every year.

###  HPV Polymerase Chain Reaction and Genotyping

 Genomic DNA from LBC samples of 1214 women was isolated using the G-spin Total DNA Extraction kit (iNtRON Biotechnology, Seongnam, Korea) according to the manufacturer’s instructions. The isolated DNA was preserved at -20 °C for later analysis with polymerase chain reaction (PCR). An HPV PCR kit (Pars Tous Biotechnology, Mashhad, Iran) was used to determine the presence of HPV in the samples. The kit contains HPV-specific universal primers that amplify a 450 bp fragment of the virus genome. The kit’s internal control consists of primers that amplify a 250 bp fragment of a human housekeeping gene. PCR was performed in 40 cycles with three steps of denaturation at 94 °C for 30 seconds, annealing at 55 °C for 30 seconds, and extension at 72 °C for 30 seconds. Distilled water was used as a sample for PCR negative control and the HPV DNA sample in the kit was used as a PCR positive control. To investigate the amplification of a 450 bp fragment, 10 μL of the PCR product was electrophoresed on 2% agarose gel, and the presence of 450 bp bands was examined by gel documentation. Since in some cases, HPV genotyping was not further requested by the patient’s physician, only 277 of the HPV PCR positive samples were examined for HPV genotyping. During the different time periods of the study, two kits, AMPLIQUALITY HPV-TYPE EXPRESS v3.0 (AB Analitica, Padova, Italy) and PapillomaStrip (Operon, Zaragoza, Spain), were used for genotyping following the manufacturer’s instructions, which are based on Single-Step PCR and Reverse Line Blot. The AMPLIQUALITY HPV-TYPE EXPRESS v3.0 kit can detect the following 40 HPV genotypes: hrHPV 16, 18, 31, 33, 35, 39, 45, 51, 52, 56, 58, 59, 66, 68; lrHPV 6, 11, 26, 40, 42, 43, 44, 53, 54, 55, 61, 62, 64, 67, 69, 70, 71, 72, 73, 81, 82, 83, 84, 87, 89, 90. The PapillomaStrip test is able to detect 37 HPV genotypes: hrHPV 16, 18, 26, 31, 33, 35, 39, 45, 51, 52, 53, 56, 58, 59, 66, 68, 69, 73 and 82; lrHPV 6, 11, 40, 42, 43, 44, 54, 61, 62, 67, 70, 71, 72, 74, 81, 83, 84 and 91.

###  Statistical Analysis

 Data were analyzed using SPSS Version 16 (SPSS, Chicago, IL, USA). Frequency tables were produced for HPV genotypes including hrHPV, lrHPV and lrHPV-hrHPV-mixed genotypes. We used chi-square to evaluate the presence of a statistically significant relationship between two nominal variables, and the phi coefficient was employed to assess the effect size. The chi-square test was valid if at least 80% of the expected frequencies were five or larger. Kendall tau-b was used for detecting trends. *P* values < 0.05 were considered statistically significant.

## Results

###  Overall HPV Prevalence and Genotype Distribution

 Following a 5-year study of 1214 women for HPV detection by PCR, 386 women were positive for HPV infection; thus, the total HPV prevalence was 31.8% for all genotypes (Table 1). The annual survey showed that the HPV prevalence increased annually from 2015 to 2020, with a medium effect size (*P* < 0.001, Kendall tau-b = 0.493). The prevalence of HPV-positive samples in April 2019 - March 2020 showed a 14-fold increase compared to April 2015 - March 2016, with a large effect size (*P* < 0.001, phi = 0.693).

**Table 1:** Annual Distribution of HPV Prevalence in 1214 Tested Women

**Year**	**Positive PCR**		**Negative PCR**		**Total**
	**No.**	**% (95% CI)**	**No.**	**% (95% CI)**	
Apr 2015–Mar 2016	15	5.1 (2.9-8.2)	281	94.9 (91.8-97.1)	296
Apr 2016–Mar 2017	70	15.8 (12.6-19.6)	372	84.2 (80.4-87.4)	442
Apr 2017–Mar 2018	60	48 (38-57.1)	65	52 (42.9-61)	125
Apr 2018–Mar 2019	78	63.9 (54.8-72.4)	44	36.1 (27.6-45.3)	122
Apr 2019–Mar 2020	163	71.2 (64.9-76)	66	28.8 (23.1-35.2)	229
Total	386	31.8 (29.2-34.5)	828	68.2 (65.5-70.8)	1214

 At the request of physicians, 277 of the PCR-positive samples were tested for HPV genotype; 37 HPV genotypes were detected, among which HPV 6 (9.1%), 53 (4.7%), 31 (3%), 42 (2.8%), 16 (2.5%), 51 (2.2%), 18 (2%), and 66 (1.8%) had the highest frequency (Table 2). The frequency of HPV 6 was 24% of the total frequency of the 37 detected genotypes.

**Table 2:** Annual Prevalence of HPV Genotypes in 1214 Tested Women

	**Genotype**	**Apr 2015 - Mar 2016**		**Apr 2016 - Mar 2017**		**Apr 2017 - Mar 2018**		**Apr 2018 - Mar 2019**		**Apr 2019 - Mar 2020**		**Total**	
		**Positive No.**	**Prevalence, % (95% CI)**	**Positive No.**	**Prevalence, % (95% CI)**	**Positive No.**	**Prevalence, % (95% CI)**	**Positive No.**	**Prevalence, % (95% CI)**	**Positive No.**	**Prevalence, % (95% CI)**	**Positive No.**	**Prevalence, % (95% CI)**
hrHPV genotype	HPV 31	0	0	0	0	2	1.6 (0.2-5.7)	19	15.6 (9.6-23.3)	16	7 (4.1-11.1)	37	3 (2.1-4.2)
	HPV 16	2	0.7 (0.1-2.4)	7	1.6 (0.6-3.2)	3	2.4 (0.5-6.9)	6	4.9 (1.8-10.4)	12	5.2 (2.7-8)	30	2.5 (1.7-3.5)
	HPV 51	0	0	6	1.4 (0.5-2.9)	4	3.2 (0.9-8)	9	7.4 (3.4-13.5)	8	3.5 (1.5-6.8)	27	2.2 (1.5-3.2)
	HPV 18	4	1.4 (0.4-3.4)	5	1.1 (0.4-2.6)	3	2.4 (0.5-6.9)	6	4.9 (1.8-10.4)	6	2.6 (1-5.6)	24	2 (1.3-2.9)
	HPV 66	0	0	1	0.2 (0.01-1.3)	1	0.8 (0.02-4.4)	8	6.6 (2.9-12.5)	12	5.2 (2.7-8)	22	1.8 (1.1-2.7)
	HPV 39	0	0	1	0.2 (0.01-1.3)	2	1.6 (0.2-5.7)	5	4.1 (1.3-9.3)	11	4.8 (2.4-8.4)	19	1.6 (0.9-2.4)
	HPV 52	0	0	2	0.5 (0.1-1.6)	1	0.8 (0.02-4.4)	1	0.8 (0.02-4.5)	15	6.6 (3.7-10.6)	19	1.6 (0.9-2.4)
	HPV 45	0	0	2	0.5 (0.1-1.6)	4	3.2 (0.9-8)	7	5.7 (2.3-11.5)	5	2.2 (0.7-5)	18	1.5 (0.9-2.3)
	HPV 68	0	0	2	0.5 (0.1-1.6)	1	0.8 (0.02-4.4)	5	4.1 (1.3-9.3)	6	2.6 (1-5.6)	14	1.2 (0.6-1.9)
	HPV 56	0	0	2	0.5 (0.1-1.6)	0	0	4	3.3 (0.9-8.2)	7	3.1 (1.2-6.2)	13	1.1 (0.6-1.8)
	HPV 58	0	0	2	0.5 (0.1-1.6)	6	4.8 (1.8-10.2)	1	0.8 (0.02-4.5)	2	0.9 (0.1-3.1)	11	0.9 (0.4-1.6)
	HPV 59	0	0	1	0.2 (0.01-1.3)	1	0.8 (0.02-4.4)	1	0.8 (0.02-4.5)	7	3.1 (1.2-6.2)	10	0.8 (0.4-1.5)
	HPV 35	0	0	0	0	0	0	0	0	2	0.9 (0.1-3.1)	2	0.2 (0.02-0.6)
lrHPV genotype	HPV 6	0	0	16	3.6 (2.1-5.8)	21	16.8 (10.7-24.5)	17	13.9 (8.3-21.4)	57	24.9 (19.4-31)	111	9.1 (7.6-10.9)
	HPV 53	0	0	15	3.4 (1.9-5.5)	20	16 (10.1-23.6)	14	11.5 (6.4-18.5)	8	3.5 (1.5-6.8)	57	4.7 (3.6-6)
	HPV 42	0	0	0	0	0	0	22	18 (11.7-26)	12	5.2 (2.7-8)	34	2.8 (1.9-3.9)
	HPV 11	0	0	3	0.7 (0.14-1)	2	1.6 (0.2-5.7)	6	4.9 (1.8-10.4)	10	4.4 (2.1-7.9)	21	1.7 (1.1-2.6)
	HPV 62	0	0	0	0	3	2.4 (0.5-6.9)	3	2.5 (0.5-7)	8	3.5 (1.5-6.8)	14	1.2 (0.6-1.9)
	HPV 54	0	0	0	0	1	0.8 (0.02-4.4)	4	3.3 (0.9-8.2)	9	3.9 (1.8-7.3)	14	1.2 (0.6-1.9)
	HPV 43	0	0	0	0	1	0.8 (0.02-4.4)	2	1.6 (0.2-5.8)	6	2.6 (1-5.6)	9	0.7 (0.3-1.4)
	HPV 82	0	0	3	0.7 (0.14-1)	2	1.6 (0.2-5.7)	1	0.8 (0.02-4.5)	3	1.3 (0.3-3.8)	9	0.7 (0.3-1.4)
	HPV 40	0	0	0	0	3	2.4 (0.5-6.9)	3	2.5 (0.5-7)	3	1.3 (0.3-3.8)	9	0.7 (0.3-1.4)
	HPV 61	0	0	0	0	2	1.6 (0.2-5.7)	1	0.8 (0.02-4.5)	5	2.2 (0.7-5)	8	0.7 (0.3-1.3)
	HPV 91	0	0	0	0	0	0	3	2.5 (0.5-7)	5	2.2 (0.7-5)	8	0.7 (0.3-1.3)
	HPV 81	0	0	0	0	0	0	1	0.8 (0.02-4.5)	5	2.2 (0.7-5)	6	0.5 (0.2-1.1)
	HPV 84	0	0	0	0	0	0	4	3.3 (0.9-8.2)	2	0.9 (0.1-3.1)	6	0.5 (0.2-1.1)
	HPV 89	0	0	2	0.5 (0.1-1.6)	1	0.8 (0.02-4.4)	1	0.8 (0.02-4.5)	0	0	4	0.3 (0.1-0.8)
	HPV 26	0	0	0	0	2	1.6 (0.2-5.7)	1	0.8 (0.02-4.5)	1	0.4 (0.01-2.4)	4	0.3 (0.1-0.8)
	HPV 67	0	0	0	0	0	0	0	0	3	1.3 (0.3-3.8)	3	0.2 (0.1-0.7)
	HPV 73	0	0	0	0	0	0	0	0	2	0.9 (0.1-3.1)	2	0.2 (0.02-0.6)
	HPV 90	0	0	0	0	0	0	0	0	2	0.9 (0.1-3.1)	2	0.2 (0.02-0.6)
	HPV 74	0	0	0	0	0	0	1	0.8 (0.02-4.5)	1	0.4 (0.01-2.4)	2	0.2 (0.02-0.6)
	HPV 83	0	0	0	0	0	0	0	0	1	0.4 (0.01-2.4)	1	0.1 (0-0.5)
	HPV 44	0	0	0	0	1	0.8 (0.02-4.4)	0	0	0	0	1	0.1 (0-0.5)
	HPV 70	0	0	0	0	0	0	1	0.8 (0.02-4.5)	0	0	1	0.1 (0-0.5)
	HPV 87	0	0	0	0	0	0	0	0	1	0.4 (0.01-2.4)	1	0.1 (0-0.5)
	HPV 69	0	0	0	0	0	0	0	0	1	0.4 (0.01-2.4)	1	0.1 (0-0.5)

###  Distribution of hrHPV and lrHPV Genotypes

 During the five years of the study, 13 hrHPV and 24 lrHPV genotypes were detected. Details of the distribution of hrHPV and lrHPV genotypes are shown in Table 2. HPV 31 (3%) was the most prevalent hrHPV, followed by HPV 16 (2.5%) and 51 (2.2%). Among the lrHPV genotypes, HPV 6 (9.2%) was the most common, followed by HPV 53 (4.7%) and 42 (2.8%).

 The annual prevalence of the hrHPV genotypes HPV 18 and 58; and the lrHPV genotype HPV 53 had a decreasing trend during the study (*P* < 0.05). However, the annual prevalence trend of the hrHPV genotypes HPV 31 and 52; and the lrHPV genotypes HPV 54 and 81 were increasing during the study (*P* < 0.05). Among the hrHPV genotypes, HPV 35 was detected only in the last year of the study. The lrHPV genotypes including HPV 67, 73, 90, 83, 87, and 69 were detected in the last year of the study.

###  Single and Multiple HPV Infections

 The range of multiple infections varied between two to eight genotypes (Table 3). The annual prevalence trend was decreasing for triple infections (*P* < 0.01).

**Table 3:** Annual Distribution of Single and Multiple Infections in 1214 Tested Women

**Genotype**	**Apr 2015 - Mar 2016**		**Apr 2016 - Mar 2017**		**Apr 2017 - Mar 2018**		**Apr 2018 - Mar 2019**		**Apr 2019 - Mar 2020**		**Total**	
	**Positive No.**	** Prevalence, % (95% CI)**	**Positive No.**	** Prevalence, % (95% CI)**	**Positive No.**	** Prevalence, % (95% CI)**	**Positive No.**	** Prevalence, % (95% CI)**	**Positive No.**	** Prevalence, % (95% CI)**	**Positive No.**	** Prevalence, % (95% CI)**
Single	6	2 (0.8-4.4)	11	2.5 (1.3-4.4)	22	17.6 (11.4-25.4)	25	20.5 (13.7-28.8)	62	27.1 (21.4-33.3)	126	10.4 (8.7-12.2)
Double	0	0	8	1.8 (0.8-3.5)	12	9.6 (5.1-16.2)	23	18.9 (12.3-26.9)	30	13.1 (9-18.2)	73	6 (4.7-7.5)
Triple	0	0	8	1.8 (0.8-3.5)	7	5.6 (2.3-11.2)	14	11.5 (6.4-18.5)	9	3.9 (1.8-7.3)	38	3.1 (2.2-4.3)
Quadruple	0	0	2	0.5 (0.1-1.6)	3	2.4 (0.5-6.9)	7	5.7 (2.3-11.5)	10	4.4 (2.1-7.9)	22	1.8 (1.1-2.7)
Quintuple	0	0	1	0.2 (0.01-1.3)	0	0	2	1.6 (0.2-5.8)	5	2.2 (0.7-5)	8	0.7 (0.3-1.3)
Sextuple	0	0	1	0.2 (0.01-1.3)	0	0	0	0	4	1.7 (0.5-4.4)	5	0.4 (0.1-1)
Septuple	0	0	0	0	0	0	1	0.8 (0.02-4.5)	1	0.4 (0.01-2.4)	2	0.2 (0.02-0.6)
Octuple	0	0	0	0	1	0.8 (0.02-4.4)	0	0	1	0.4 (0.01-2.4)	2	0.2 (0.02-0.6)

 Multiple HPV infections (12.4%) were the more common type of infection among the studied population compared to single HPV infections (10.4%) (*P* < 0.05). We found that the prevalence of HPV decreased with the increasing involvement of individuals with different HPV genotypes (*P* < 0.001) (Table 3).

 The prevalence rates of single and multiple HPV infections in April 2017 - March 2018 and April 2019 - March 2020 were close to each other; however, the prevalence rates of multiple HPV infections in April 2016 - March 2017 and April 2018 - March 2019 were 1.8 and 1.9 times higher than single HPV infections, respectively (*P* < 0.05).

 As shown in Table 4, a higher number of women (13.5%) were infected with one or more hrHPVs (including hrHPV-only and lrHPV-hrHPV-mixed infections), compared to 113 individuals (9.3%) who were infected with lrHPV-only (*P* < 0.01). However, lrHPV-only cases (9.3%) were more prevalent than hrHPV-only cases (6.2%) (*P* < 0.01).

**Table 4:** Annual Distribution of hrHPV-only, lrHPV-only, and lrHPV-hrHPV-Mixed Infections in 1214 Tested Women

**Year**	**hrHPV-only**		**lrHPV-only**		**lrHPV-hrHPV-Mixed**	
	**Positive No.**	** Prevalence, % (95% CI)**	**Positive No.**	** Prevalence, % (95% CI)**	**Positive No.**	** Prevalence, % (95% CI)**
Apr 2015 - Mar 2016	6	2 (0.8-4.4)	0	0	0	0
Apr 2016 - Mar 2017	3	0.7 (0.1-1)	13	2.9 (1.6-4)	15	3.4 (1.9-5.5)
Apr 2017 - Mar 2018	9	7.2 (3.3-13.2)	23	18.4 (11.4-25.4)	13	10.4 (5.7-17.1)
Apr 2018 - Mar 2019	18	14.8 (9-22.3)	23	18.9 (12.3-26.9)	31	25.4 (17-34.1)
Apr 2019 - Mar 2020	39	17 (12.4-22.5)	54	23.6 (18.2-29.6)	30	13.1 (9-18.2)
Total	75	6.2 (4.9-7.7)	113	9.3 (7.7-11.1)	89	7.3 (5.9-8.9)

 The prevalence of lrHPV-hrHPV-mixed infections slightly decreased during the study (*P* = 0.054).

###  Prevalence of Genotypes in Single and Multiple HPV Infections

**Figure 1:**
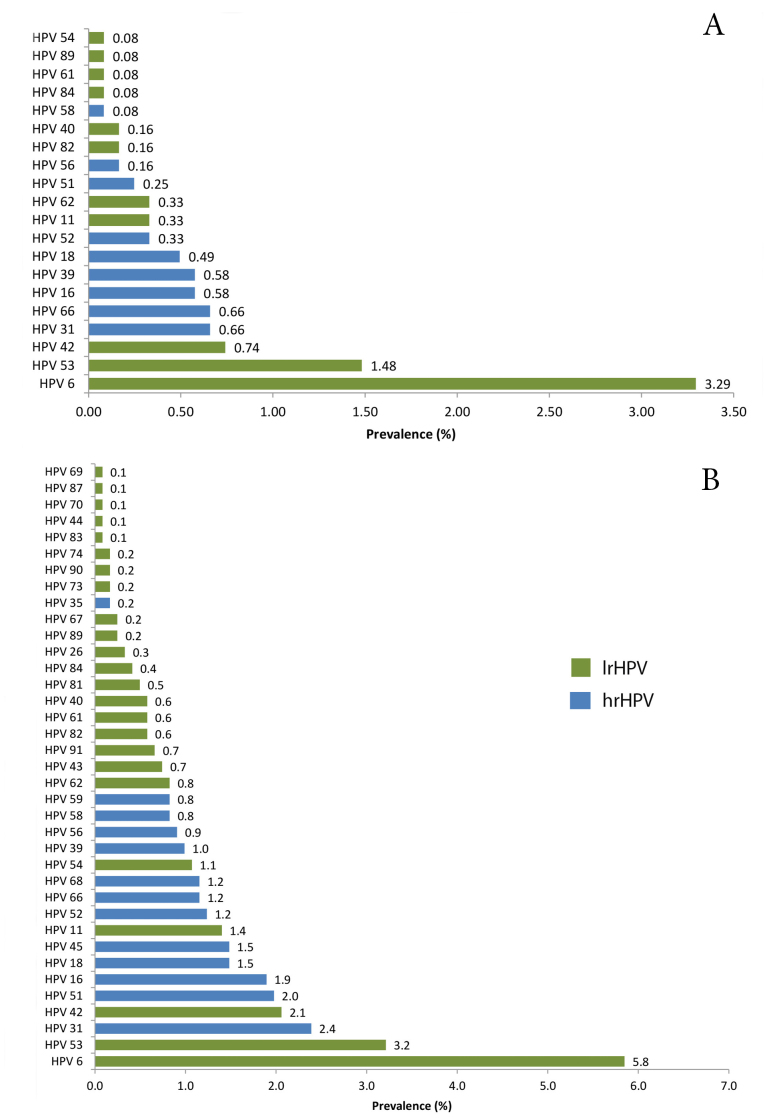
Prevalence of lrHPV and hrHPV Genotypes in Single (A) and Multiple (B) HPV Infections in 1214 Tested Women

 The genotype prevalence for single and multiple HPV infections is shown in Figure 1. HPV 6, 53, and 42 were the most prevalent lrHPV genotypes in single and multiple HPV infections; however, their prevalence in multiple HPV infections was respectively 1.8, 2.2, and 2.8 times higher than in single HPV infections (*P* < 0.05).

 HPV 31 was the most prevalent hrHPV genotype in single and multiple HPV infections. The prevalence was variable between single and multiple HPV infections for other prevalent hrHPV genotypes, especially for HPV 51 (*P* < 0.001) (Figure 1).

 As with lrHPV genotypes, the prevalence of hrHPV genotypes in multiple HPV infections was higher than in single HPV infections. The prevalence of HPV 31, 51, and 16 in multiple HPV infections was respectively 3.6, 8, and 3.3 times higher than in single HPV infections (*P* < 0.05).

 The HPV 35, 45, 59, 68, 43, 73, 81, 83, 90, 67, 44, 91, 70, 26, 74, 87 and 69 genotypes were present only in multiple HPV infections. In multiple and single HPV infections, 37.8% and 45% of infections were caused by hrHPV, respectively.

## Discussion

 Research on the detection and prevalence of HPV genotypes over the years is crucial for designing screening programs for cervical cancer diagnosis and HPV vaccine efficacy evaluation in women. Annual efforts are made around the world to provide epidemiological information on the prevalence of HPV genotypes by molecular methods. For this purpose, we conducted one of the largest studies in terms of sample size and duration in Iran.

 The results of this molecular study that was performed on 1214 women over five years showed that the overall prevalence of HPV in women in Mashhad was 31.8%. The annual study showed that the annual prevalence of HPV-positive women increased from 2015 to 2020, and HPV genotypes had variable annual prevalence. Accordingly, multi-year studies of the virus prevalence, even in a particular geographical location, are required to provide a general picture of HPV prevalence. Regarding the variability of the annual HPV prevalence, the special conditions of Mashhad city should be considered, including its positions as the capital city of the province, resulting in patient referral from other cities to Mashhad medical centers; and annual travel of several million pilgrims from other Iranian cities or Muslim countries to Mashhad.^16^

 In recent Iranian studies on cervical fluid samples for HPV DNA detection, the HPV prevalence ranged from 2.4 to 52% with an average of 27.8%.^11-15,17-20^ The results of an Iranian meta-analysis showed that the prevalence of HPV in Iranian women with normal cervical cytology was 9%.^21^ Considering that the global prevalence of HPV in women varies from 2 to 44%, the overall prevalence rate of 31.8% indicates the high prevalence of HPV in Mashhad compared to the national and global HPV prevalence.^22,23^ A worldwide meta-analysis study showed that the HPV prevalence in women with normal cervical cytology was 10.4%. Accordingly, the highest prevalence of HPV was found in Africa (22.1%), followed by Central America and Mexico (20.4%) and North America (11.3%), and to a lesser extent in Europe (8.1%) and Asia (8.0%).^24^

 The distribution of different HPV genotypes in women varies depending on the geographical area and health status. In this study, HPV 6 (9.1%), 53 (4.7%), 31 (3%), 42 (2.8%), and 16 (2.5%) were five prevalent HPV genotypes in women.

 In a study by Farahmand et al, HPV 6 was the most prevalent genotype in Iranian women with a frequency of 9.3%, which is similar to our study. However, HPV 11 (2.3%), 16 (1.8%) and 18 (1.2%) occupied the next ranks in terms of prevalence.^13^ In a study on 1133 women in Tehran from 2012 to 2015, there were similarities with our study in terms of the detected genotypes; HPV 6 (25.1%), 16 (9.8%), 31 (6.4%), and 53 (5.9%) were the most prevalent genotypes.^25^ In a study on samples taken from 31 provinces in Iran, HPV 6, 16, 11, and 52/53 were the most common genotypes detected in women.^17^ A meta-analysis reported the HPV 6 along with HPV 11 and 42 as the prevalent lrHPV genotypes in Iran.^21^ HPV 6 has been also detected as the most common lrHPV genotype in the Americas; however, a lower prevalence of the virus has been reported in Asia.^26^ Although HPV 6 and 11 (the ninth genotype detected in this study) are classified as lrHPV genotypes, they cause genital warts, which include a wide variety of skin and mucosal lesions. Fortunately, it was shown that the implementation of extensive vaccination with a quadrivalent HPV vaccine (includes HPV 6 and 11) can significantly prevent the development of genital warts in young women.^27^

 We found that HPV 31 (3%), 16 (2.5%), 51 (2.2%), 18 (2%), and 66 (1.8%) were the most prevalent hrHPV genotypes among the 13 hrHPV genotypes detected in the present study. HPV 16 and 18, which respectively ranked second and fourth in the prevalence of hrHPV genotypes in this study, are globally the most prevalent oncogenic genotypes in the female population and patients with cervical cancer or its precursors.^24,28,29^ In a study to evaluate the performance of liquid-based cytology compared to genotyping for cervical cancer screening, it was found that the detection of HPV 16 and 18 could be a more efficient and sensitive alternative to cytology for cervical cancer screening.^30^ Accordingly, considering that qualified cytopathologists are needed to implement a cytology-based cervical cancer prevention program, detecting hrHPV genotypes can be a good alternative to cytology, especially in a developing country like Iran.

 HPV 31 was the most prevalent hrHPV in the present study. In a study to evaluate the ability of vaccines Gardasil^®^ (HPV-6/11/16/18, Merck) and Cervarix^®^ (HPV-16/18, GlaxoSmithKline Biologicals, GSK) to induce cross-neutralizing antibodies against HPV 31, 33, and 45, it was found that these vaccines can induce cross-reactive immunity against these genotypes. The induced antibody titers were higher in women, especially in relation to HPV 31 and 45, than men.^31^ Given that HPV 31 and HPV 45 were the first and eighth most common hrHPV genotypes in this study, it can be hoped that these vaccines will be able to prevent these infections.

 We detected HPV 51 as the third most prevalent hrHPV genotype with a difference of 0.3% after HPV 16. HPV 51 is known as a hrHPV genotype in the cervical intraepithelial neoplasia (CIN), and its prevalence varies from 0 to 2.3% in different countries.^32^ A study in Italy found that the prevalence of HPV 16 and 51 was directly related to the severity of cervical lesions and that HPV 51 was involved in the pathogenesis of invasive cervical cancer.^33^ In a study conducted in Spain on a cytological sample of 67 935 females over a 4-year period, HPV 16 (60.9%) and 51 (51.7%) were the most prevalent genotypes.^34^ Unfortunately, there is evidence that vaccines Gardasil^®^ and Cervarix^®^ might not have the efficacy to protect against HPV 51. In a 19-year-old woman who received the full vaccination of Gardasil^®^ (HPV6/11/16/18 vaccine), HPV 51 infection was detected one year after receiving the vaccine.^35^ In a study performed to confirm the HPV 51 prevalence that was observed in a Base HPV 2009 study population, the results showed a high prevalence for HPV 51. It was suggested that HPV 51 should be considered in the manufacture of multivalent HPV vaccines.^36^ Studies also showed that phylogenetic relatedness is important in the development of cross-protection, and because HPV 51 (A5 species) is phylogenetically different from HPV 16 and 18 (A9 species), the present commercial vaccines might not provide enough cross-protection against HPV 51.^37,38^ In a number of Iranian studies, HPV 51 has been detected; however, in our study, HPV 51 was relatively a highly prevalent hrHPV genotype.^12,14,21,39^

 HPV 66 (1.8%) was the fifth most common hrHPV in our study. A study on Mexican women found a high prevalence of HPV 66 (32.8%) and in spite of hrHPV classification, suggested that HPV 66 could be associated with lesions that will not progress to cancer.^40^ A study in Thailand also found that HPV 66 (6.9%) is one of the most common genotypes in women.^41^ In some Iranian studies, HPV 66 has been detected as one of the most prevalent genotypes in women.^11,17^ In a study in northern Iran, HPV 66 was the third most common genotype after HPV 16 and 6.^11^ In an Iranian meta-analysis, HPV 66 was the fourth and fifth most common genotype in women with normal cervix and ASCUS, respectively.^21^

 Three prevalent lrHPV genotypes, including HPV 6 (9.2%), 53 (4.7%), and 42 (2.8%) that were detected in this study, are prevalent HPV genotypes in Iran.^21^ HPV 6 was also the most prevalent lrHPV genotype detected among Americans (2.9%).^26^ Considering that commercial vaccines provide protection against HPV 6, the high prevalence of HPV 6 in this study is expected due to the low coverage of HPV vaccination in Mashhad.

 In this study, a higher number of women had multiple HPV infections (12.4%) compared to single HPV infections (10.4%). Studies have shown that multiple infections were more common in young women than in older ones.^42,43^ Young women are more sexually active and their immune system is less sensitive to HPV genotypes, so they are more likely to become co-infected with several genotypes. However, the majority of HPV infections in young women are transient and the infection may be cleared or be dominated by one or two HPV genotypes.^44,45^ Due to lack of information on the age of the subjects, we cannot determine the relationship between age and multiple infections in this study. However, the high number of multiple infections (12.4%) can indicate a high circulation of different HPV genotypes in Mashhad. Multiple HPV infections can increase the duration of HPV infection and the risk of cervical cancer, and in this regard, the high prevalence of multiple infections found in this study should be considered for the implementation of effective preventive measures.^46^

 In conclusion, considering the high prevalence of multiple and single HPV infections in women in Mashhad, the need for high-coverage vaccination should be emphasized. Unfortunately, HPV vaccination expenses are not supported by our government and HPV vaccination is not mandatory. The relatively high cost of available commercial HPV vaccines and lack of women’s awareness about the risks of HPV infection have led to very low coverage of HPV vaccination in Iran. In addition, the presence of HPV genotypes such as HPV 51, which is one of the most prevalent hrHPV genotypes detected in this study, adds to this problem because the available vaccines in Iran such as Gardasil^®^ might not be able to induce cross-protection against this genotype. Finally, it is recommended that cervical cancer screening programs for the initial diagnosis of hrHPV should be implemented in Mashhad and that HPV vaccination is included in national disease prevention programs, and the vaccination costs are covered by the government.
